# Fabrication of Biphasic Hydroxyapatite/Calcium Sulfate Blocks via Hydrothermal Partial Phase Transformation for Bone Substitutes

**DOI:** 10.1055/s-0046-1816076

**Published:** 2026-02-10

**Authors:** Sunarso Sunarso, Gita Andani Pradana, Syifaa Sagakirana, Ellyza Herda, Azizah Intan Pangesty

**Affiliations:** 1 Department of Dental Materials Science, Faculty of Dentistry, Universitas Indonesia, Jakarta, Indonesia; 2 Undergraduate Program, Faculty of Dentistry, Universitas Indonesia, Jakarta, Indonesia; 3 Department of Metallurgical and Material Engineering, Faculty of Engineering, Universitas Indonesia, Depok, Indonesia

**Keywords:** biphasic HA/CS, bone substitute, cytotoxicity, diametral tensile strength, hydrothermal conversion, solubility

## Abstract

**Objective:**

This article aims to fabricate, characterize, and evaluate the mechanical strength and cytotoxicity of biphasic hydroxyapatite/calcium sulfate (HA/CS) blocks using partial hydrothermal conversion of gypsum blocks.

**Materials and Methods:**

Gypsum blocks were immersed in Na _3_ PO _4_ solution under hydrothermal conditions at 150 °C for 6, 12, and 24 hours, denoted as 6-HA/CS, 12-HA/CS, and 24-HA/CS, respectively. After that, the phase composition was analyzed using X-ray diffraction. Diametral tensile strength (DTS), solubility in Tris-HCl buffer, and cytotoxicity on cell cultures were measured.

**Results:**

Hydrothermal treatment produced biphasic HA/CS with increasing hydroxyapatite content. All HA/CS groups exhibited a significant reduction in DTS (∼0.5–0.7 MPa) compared with gypsum (2.06 MPa; *p* < 0.05). Solubility was lower in HA/CS than in gypsum (206.15 mg/L), with the 24-HA/CS group showing the lowest value (94.70 mg/L). Cytotoxicity testing demonstrated cell viability above 70% for all groups, meeting ISO standards of non-cytotoxicity.

**Conclusion:**

Partial hydrothermal conversion of gypsum block successfully produced biphasic HA/CS with tunable phase composition, reduced solubility, and acceptable cytotoxicity.

## Introduction

Hydroxyapatite (HA) has been known as a bone graft material for years. 1 2 3 It is biocompatible and has a composition close to that of human bone. 4 Nevertheless, its slow resorption rate during implantation is the main drawback. 5 Studies have reported that HA remained at the implantation site even years after implantation. 6 Several attempts have been made to improve the resorbability of HA, including combination with faster resorbing calcium phosphate ceramics to form biphasic calcium phosphate (BCP) materials. BCP that is composed of HA and β-tricalcium phosphate (HA/β-TCP) demonstrated improved resorbability. 7 8 β-TCP crystals have higher solubility compared with HA. Therefore, a combination of HA and β-TCP enhances resorption during implantation. Despite good clinical results, biphasic HA/β-TCP exhibits moderate resorption, which may be insufficient when faster bone formation is required. Moreover, β-TCP is considered to have a relatively high cost, which limits its accessibility. 9

Biphasic HA/calcium sulfate (HA/CS) has emerged as a promising alternative bone graft material, providing faster resorption compared with BCP. 10 11 The solubility of CS is higher compared with β-TCP. HA/CS has been used as a bone filler for decades and is known to be completely resorbed. 12 13 Conventional biphasic HA/CS materials are usually fabricated through high-temperature sintering. 10 This fabrication method is costly and results in highly crystalline HA, which exhibits poor resorbability when implanted. 5 Previous studies have shown that sintered HA is hardly resorbed by osteoclasts. 14 15 16 Therefore, there is a growing need for alternative fabrication methods that operate at significantly lower temperatures and yield more bioresorbable materials.

In our earlier work, we successfully synthesized non-sintered HA using a dissolution–precipitation process under hydrothermal conditions. 17 Building on that, we modified the previously developed hydrothermal method to produce biphasic HA/CS without the need for sintering. Therefore, this study aimed to fabricate biphasic HA/CS through partial hydrothermal conversion of gypsum, to evaluate how conversion conditions influence the resulting phase composition, to determine the DTS of the obtained biphasic HA/CS, and to measure the solubility in Tris-HCl buffer solution to simulate the physiological conditions. We also evaluated the cytotoxicity of the fabricated biphasic HA/CS.

## Materials and Methods

### Sample Preparation

Gypsum block (Gyp) was prepared by mixing CS hemihydrate powder (Wako Chemical, Japan) at a powder-to-liquid ratio of 1:2. The paste was then molded into an acrylic split mold with a diameter of 6 mm and a height of 3 mm. The paste was allowed to set for 24 hours at room temperature. After setting, the set blocks were removed from the acrylic split mold and immersed in 0.5 mol/L Na _3_ PO _4_ .12H _2_ O (Merck, Germany) solution, then placed in a hydrothermal vessel. The hydrothermal vessel is composed of a stainless-steel outer shell and an inner chamber made of polytetrafluoroethylene (PTFE). The vessel was subsequently placed in an oven at 150 °C for 6, 12, and 24 hours, denoted as 6-HA/CS, 12-HA/CS, and 24-HA/CS, respectively.

### Material Characterization

X-ray diffraction (XRD) analysis (PANalytical X'Pert Pro; Cu Kα, *λ* = 1.54 Å) was used to evaluate the phase composition of the obtained biphasic HA/CS blocks. Phase composition was analyzed by Rietveld refinement of the XRD data. The refinement was performed using X'Pert HighScore software with an automatic Rietveld profile employing Pseudo-Voigt fitting. XRD was operated at 30 mA and 40 kV with a step size of 0.0170°, covering a 2θ range from 10.0084° to 89.9764°.

### Mechanical Strength Test

The diametral tensile strength (DTS) of the obtained biphasic HA/CS blocks and the set gypsum blocks was measured using a universal testing machine (Shimadzu AGSX-50kN). A 5,000 N load cell was employed, with the crosshead speed set at 0.5 mm/min. The maximum load at which each specimen fractured was recorded. For each group, 10 specimens ( *n* = 10) were tested, and the average values were reported.

### In Vitro Solubility Test

The solubility of the biphasic HA/CS was evaluated using Tris-HCl buffer to simulate physiological conditions. 17 18 First, a Tris–HCl buffer solution was prepared by dissolving tris(hydroxymethyl)aminomethane (Merck, Germany) to a final concentration of 0.05 mol/L. The pH was adjusted to 7.4 by gradually adding concentrated hydrochloric acid (Merck, Germany) while monitoring the pH.

For the solubility test, biphasic HA/CS blocks were immersed in Tris–HCl buffer solution at a ratio of 1 mg/mL in high-density polyethylene (HDPE) bottles. The bottles were then incubated at 37 °C for 7 days. Following the immersion period, the samples were removed by filtration, and the filtrates were analyzed using atomic absorption spectroscopy (AAS) (Shimadzu AA-625–01) to quantify the calcium ion concentration. The weights of the specimens before and after immersion in Tris-HCl buffer were recorded. The difference between the initial weight and the post-immersion weight was taken as weight loss. For each composition, three specimens ( *n* = 3) were tested.

### Cytotoxicity Test

Cytotoxicity was assessed using the BHK-21 fibroblast cell line (CCL-10, ATCC), which is commonly employed in toxicity screening protocols. 19 20 21 Cells were seeded into 96-well plates (Bio-Rad) at a density of 5,000 cells per well and cultured in Dulbecco's modified eagle medium (Sigma-Aldrich) supplemented with 10% fetal bovine serum (Biosera) and a penicillin–streptomycin mix (100 U/mL and 100 μg/mL, respectively; Sigma-Aldrich). To prepare the test extracts, biphasic HA/CS materials were immersed in the culture medium at concentrations of 6.25, 12.5, 25, and 50 mg/mL and incubated at 37 °C for 24 hours. After the cells had been cultured for 24 hours, the extracts were added to the wells. On the third day, 90 μL of fresh medium was added, followed by 10 μL of MTT solution (5 mg/mL, Sigma-Aldrich). The plates were then incubated for 4 hours at 37 °C. After incubation, 100 μL of ethanol was added to each well to dissolve the resulting formazan crystals, and absorbance was recorded at 595 nm. Wells containing only the culture medium served as a control. Each experiment was tested in triplicate.

### Data Analysis

Statistical analysis was performed using one-way ANOVA to assess differences among the groups. When the ANOVA showed significant differences ( *p* < 0.05), Tukey's HSD post hoc test was applied to determine pairwise group differences. All statistical analyses were performed using KaleidaGraph.

## Results

### Biphasic HA/CS Characterization

Fig. 1 shows images taken before and after the hydrothermal process used to produce biphasic HA/CS blocks. The images indicate that the gypsum block did not collapse after the hydrothermal reactions. To analyze the phase transformation from gypsum to biphasic HA/CS, XRD characterization was performed. Fig. 2 shows the XRD pattern of the obtained biphasic HA/CS compared with that of gypsum as the precursor. XRD analysis revealed that the biphasic HA/CS displayed characteristic peaks of HA and CS anhydrate across all hydrothermal durations. We then performed compositional analysis of how much HA and CS anhydrate in the obtained biphasic HA/CS. Table 1 summarizes the phase composition of the hydrothermally treated specimens. The 6-HA/CS sample comprised 23% HA and 77% CS anhydrate. Increasing hydrothermal duration to 12 hours (12-HA/CS) and 24 hours (24-HA/CS) increased the HA content to 47 and 80%, respectively, with a corresponding reduction in CS anhydrate.

**Fig. 1:**
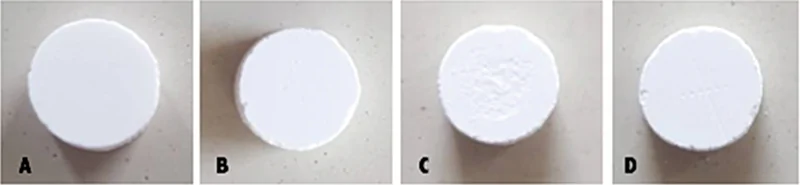
Photographs of gypsum block ( **A** ), 6-HA/CS ( **B** ), 12-HA/CS ( **C** ), and 24-HA/CS ( **D** ).

**Fig. 2:**
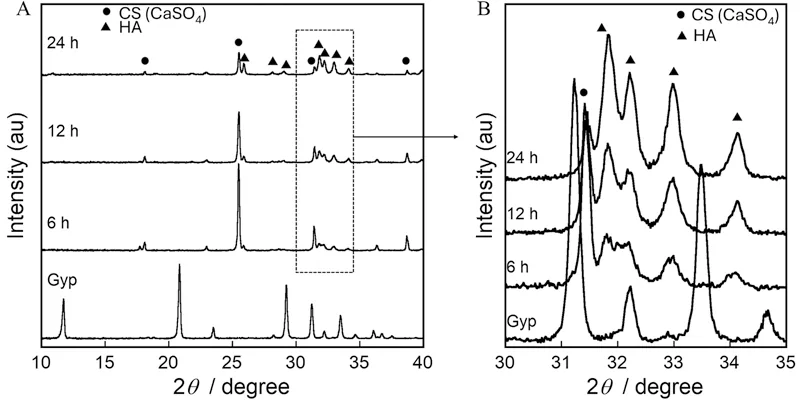
( **A, B** ) XRD diffraction of biphasic HA/CS blocks.

**Table 1:** Phase composition of biphasic HA/CS at different hydrothermal durations

Sample	Phase
Gyp	100% calcium sulfate dihydrate (CaSO _4_ .2H _2_ O)
6-HA/CS	23% hydroxyapatite
	77% calcium sulfate anhydrate (CaSO _4_ )
12-HA/CS	47% hydroxyapatite
	53% calcium sulfate anhydrate (CaSO _4_ )
24-HA/CS	80% hydroxyapatite
	20% calcium sulfate anhydrate (CaSO _4_ )

Note: Values are presented as percentages of hydroxyapatite and calcium sulfate anhydrate determined by XRD analysis.

### Mechanical Strength

To evaluate changes in mechanical strength, DTS testing was performed on biphasic HA/CS blocks and compared with the gypsum block precursor ( Fig. 3 ). The biphasic HA/CS blocks showed a roughly 75% reduction in DTS compared with the gypsum precursor, regardless of hydrothermal duration. The gypsum block precursor exhibited a DTS of 2.06 ± 0.23 MPa, which was significantly higher than that of all biphasic HA/CS groups ( *p* < 0.05). The 6-HA/CS, 12-HA/CS, and 24-HA/CS groups recorded mean values of 0.52 ± 0.18, 0.57 ± 0.14, and 0.68 ± 0.13 MPa, respectively, with no significant differences among them ( *p* > 0.05).

**Fig. 3:**
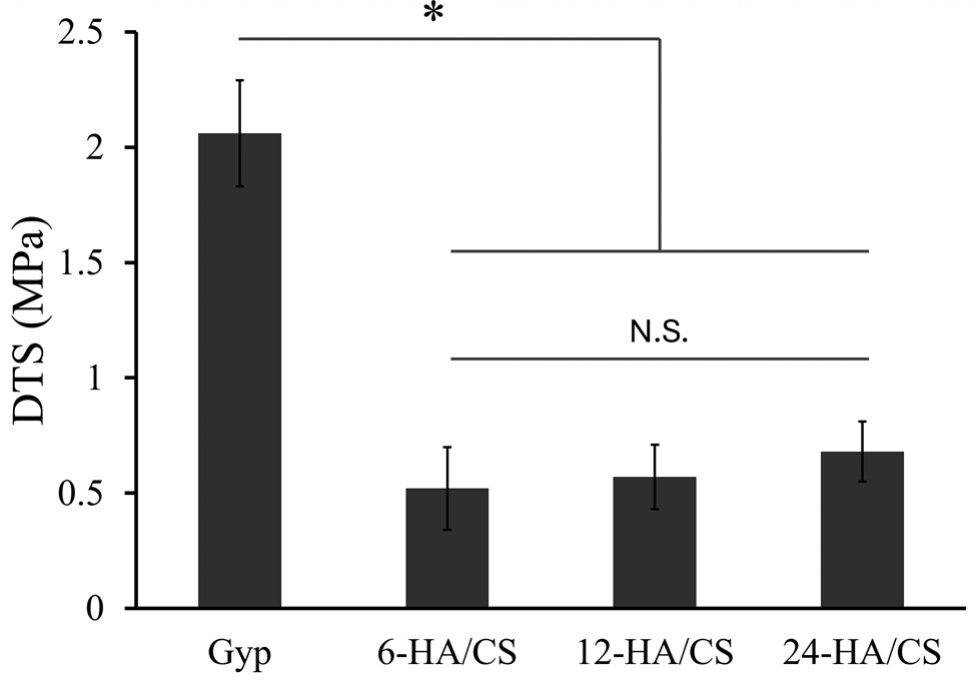
Diametral tensile strength (DTS) of biphasic HA/CS blocks compared with the gypsum block. For each group, 10 specimens were tested ( *n* = 10). The asterisk (*) denotes statistically significant differences ( *p* < 0.05), while NS indicates not significant.

### Solubility of Biphasic HA/CS

The solubility and degradation of biphasic HA/CS in Tris-HCl buffer solution are presented in Fig. 4A . The solubility of the precursor gypsum block was 206.15 ± 16.51 mg/L. After conversion into biphasic HA/CS, the solubility was significantly lower. The 24-HA/CS specimen, which had the highest HA content (80%), exhibited a solubility of 94.70 ± 2.82 mg/L, almost half that of the gypsum block. Meanwhile, the solubility values for 6-HA/CS and 12-HA/CS were 121.08 ± 8.02 mg/L and 106.99 ± 5.94 mg/L, respectively. The difference between 6-HA/CS and 24-HA/CS was statistically significant ( *p* < 0.05), whereas the differences between 6-HA/CS and 12-HA/CS and between 12-HA/CS and 24-HA/CS were not statistically significant ( *p* > 0.05).

**Fig. 4:**
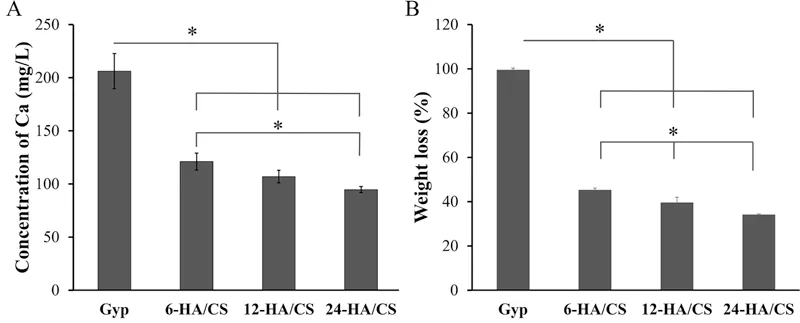
Solubility ( **A** ) and degradation ( **B** ) of biphasic HA/CS compared with gypsum after immersion in Tris-HCl buffer at 37 °C for 7 days. For each group, 3 specimens were tested ( *n* = 3). The asterisk (*) denotes statistically significant differences ( *p* < 0.05).

The degradation rate of gypsum and biphasic HA/CS was evaluated through weight loss measurements, as shown in Fig. 4B . The Gypsum block showed the highest degradation, with a weight loss of almost 100% after immersion in Tris-HCl buffer for 7 days. In comparison, all HA/CS groups exhibited significantly lower weight loss. The 6-HA/CS, 12-HA/CS, and 24-HA/CS samples degraded by 45.3 ± 0.9%, 39.6 ± 2.4%, and 34.2 ± 0.3%, respectively. Statistical analysis revealed significant differences between gypsum block and all biphasic HA/CS groups ( *p* < 0.05). Furthermore, the difference among the biphasic HA/CS groups was also significant.

### Cytotoxicity of Biphasic HA/CS

The cytotoxicity data for biphasic HA/CS are presented in Fig. 5 . All biphasic HA/CS specimens showed cell viability above 70%, which is the minimum threshold for non-cytotoxicity according to ISO standards. Raising the extract concentration to 50 mg/mL did not result in greater cytotoxicity. The cell viability of the biphasic HA/CS groups did not differ significantly, except between 6-HA/CS and 12-HA/CS, and between 24-HA/CS and 12-HA/CS.

**Fig. 5:**
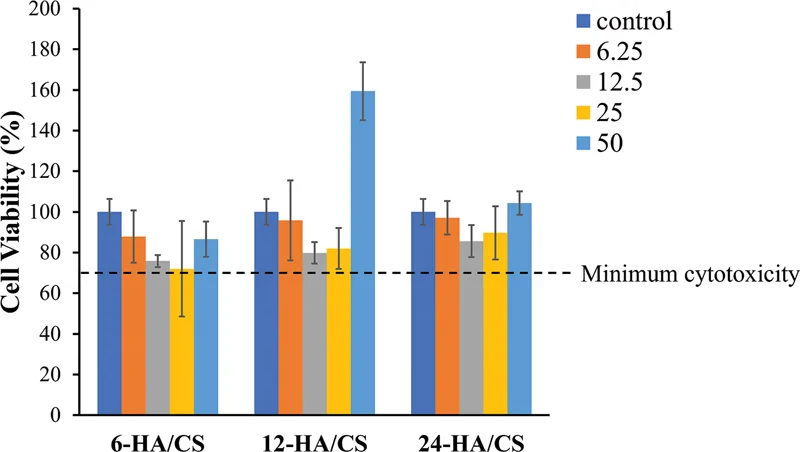
Cytotoxicity of biphasic HA/CS blocks. For each group, three specimens were tested ( *n* = 3).

## Discussion

In this study, biphasic HA/CS blocks were fabricated via an alternative approach involving partial phase transformation from gypsum block to HA/CS block through a hydrothermal process, offering a simpler and more practical way to produce the material. Using this method, the HA/CS composition can be tailored by adjusting the hydrothermal duration. For this preliminary work, three compositions were prepared with hydrothermal periods of 6, 12, and 24 hours. The first concern was whether the gypsum blocks would collapse during the hydrothermal process to form biphasic HA/CS. The results showed that the blocks kept their shape after partial phase transformation ( Fig. 1 ). The obtained biphasic HA/CS blocks were then characterized by XRD to determine their phase compositions. The XRD patterns ( Fig. 2 ) indicated that the gypsum phase has been converted into HA and CS anhydrite, with the relative proportions varying according to the hydrothermal duration ( Table 1 ). These results suggest that, in addition to the partial transformation of gypsum into HA, dehydration of gypsum also occurred, yielding CS anhydrite.

Despite retaining their original block shape, the DTS of biphasic HA/CS blocks decreased considerably compared with that of the precursor gypsum block. Similar results were observed in our previous work on the full conversion of gypsum blocks into HA, where the DTS decreased by up to 90% after hydrothermal treatment. 22 In that study, CS anhydrite was also identified as an intermediate phase before gypsum was fully transformed into pure HA. The reduction in DTS was attributed to microstructural changes in the blocks following hydrothermal processing, as we reported previously. 22 Biphasic HA/CS is intended for non–load-bearing applications where high mechanical strength is not required. The DTS values of the obtained biphasic HA/CS (∼0.5–0.7 MPa) were still adequate, falling within the range reported by several preclinical studies (∼0.1–2 MPa). 23 24

HA has been used clinically for decades. However, their slow degradation remains a major drawback. To address this limitation, HA is often combined with more rapidly resorbing materials, such as CS. 13 In this study, we measured the solubility of the obtained biphasic HA/CS in Tris-HCl buffer solution to simulate physiological conditions. Wu and Uskoković reported a correlation between the solubility and resorbability of bone graft materials. 25 In that study, the authors compared several calcium phosphate materials, such as HA, monetite, calcium pyrophosphate, and amorphous calcium phosphate, which exhibited different solubility levels. The results demonstrated that monetite, identified as the most soluble among the tested materials, exhibited the highest rate of resorption. In contrast, HA, possessing the lowest solubility, showed the lowest resorption. In this study, the solubility of biphasic HA/CS was much lower than that of gypsum ( Fig. 4 ). We did not compare its solubility directly with pure HA blocks; however, based on our previous report, the solubility of pure HA obtained via a hydrothermal process similar to the method used here was around 3 mg/L, which is very low compared with that of the biphasic HA/CS specimens. 17 This indicates that the biphasic HA/CS is likely to resorb faster upon implantation than pure HA, although this should be verified through in vivo studies.

CS has a long history of use as a bone substitute. However, some studies reported that CS can cause inflammation after implantation due to its rapid dissolution. 26 Calcium ions released from calcium-based bone substitutes play an important role in early bone healing. A moderate elevation of extracellular calcium ions can stimulate osteoblast proliferation, upregulate osteogenic markers, and enhance mineralized matrix formation. 27 In this study, we did a cytotoxicity test as a first step to see if biphasic HA/CS could cause toxic effects to cells. This simple test can give early information about its safety before doing animal or clinical studies. Based on the cytotoxicity test, all biphasic HA/CS specimens exhibited cell viability above 70%, which meets the ISO standard threshold for non-cytotoxicity. Interestingly, no clear relationship was observed between the HA/CS composition and cytotoxicity. For example, specimen 6-HA/CS, which contained 23% HA and 77% CS anhydrate, showed comparable cell viability to specimen 24-HA/CS, which contained 80% HA and 20% CS anhydrate. Therefore, a more comprehensive in vitro evaluation, including longer cultures and additional cell types such as osteoblasts, is needed to fully elucidate the biological performance of the HA/CS blocks.

Overall, these preliminary results demonstrate that biphasic HA/CS blocks can be fabricated through partial phase transformation of gypsum via a hydrothermal process while maintaining their structural integrity. The process allows varying phase composition by adjusting the hydrothermal duration, which in turn influences solubility. The obtained materials showed non-cytotoxic responses, regardless of their HA/CS ratio, suggesting potential suitability for bone graft applications where a balance between stability and resorbability is desired. Furthermore, in vitro studies, incorporating extended culture periods and relevant cell types such as osteoblasts, as well as in vivo evaluations, will be necessary to confirm their biological performance and clinical applicability.

## Conclusion

Biphasic HA/CS blocks were successfully fabricated from gypsum blocks through partial hydrothermal treatment. The obtained blocks were not collapsed after hydrothermal. The DTS decreased by ∼75% compared with that of Gypsum block precursor. XRD diffraction analysis confirmed that both HA and CS anhydrate phases exist in the block, with the HA fraction increasing alongside longer hydrothermal time. The biphasic HA/CS blocks exhibited lower solubility, particularly at higher HA contents. All biphasic HA/CS specimens demonstrated non-cytotoxic.
